# A low noise cascaded amplifier for the ultra-wide band receiver in the biosensor

**DOI:** 10.1038/s41598-021-02122-4

**Published:** 2021-11-19

**Authors:** Maissa Daoud, Mohamed Ghorbel, Hassene Mnif

**Affiliations:** 1 Research Laboratory On Electronics and Information Technologies, National Engineering School of Sfax, University of Sfax, Road Soukra, km 3.5, 3018 Sfax, Tunisia; 2 Research Laboratory On Advanced Technologies for Medicine and Signal, National Engineering School of Sfax, University of Sfax, Road Soukra, km 3.5, 3018 Sfax, Tunisia

**Keywords:** Biomedical engineering, Electrical and electronic engineering

## Abstract

This paper presents the design of an Ultra-Wide Band (UWB) Low Noise cascaded Amplifier (LNA) used for biomedical applications. The designed structure uses a technique which is based on the inductances minimization to reduce the LNA surface while maintaining low power consumption, low noise and high stability, linearity and gain. To prove its robustness, this technique was studied theoretically, optimized and validated through simulation using the CMOS 0.18 µm process. The LNA achieves a maximum band voltage gain of about 17.5 dB at [1-5] GHz frequency band, a minimum noise figure of 2 dB, IIP3 of + 1dBm and consumes only 13mW under a 2 V power supply. It is distinguished by its prominent figure of merit of 0.68.

## Introduction

Today, the passive monitoring of vital signs using biomedical sensors requires the use of wireless communication relying on the technological evolution of these devices^1–5^. Over the last decade, the scientific research in the nanotechnology field has focused on the challenges of low power requirements for medical devices to ensure a long battery pack life time^6–8^. This has become critical for surgically implanted devices where size and battery life are essential as they are implemented in highly sensitive parts of the human body such as eyes for retinal prosthesis and brain for embedded applications neurons^9,10^. In this case, the use of energy harvesting is an appropriate choice to meet the stringent power budgets^11–14^.

Several biomedical applications using Ultra Wide Band (UWB) has become essential. The "camera pills", for instance, are used as a UWB transmitter to send good quality videos outside the human body^15–17^. Other biomedical applications for the UWB can be found in^18^. The primary advantages of the UWB are the wide bandwidth and the transmitter simplicity for a UWB based Impulse Radio (IR)^19^.

Typically, the biosensor consists of a power supply unit, two transmission and reception chains, and a data processing unit. In a receiver front-end, the low noise amplifier (LNA) is a critical block since it should amplify the weak signal received from the antenna with sufficient gain and little additional noise^20^. The low noise amplifier (LNA) has very stringent requirements such as gain, noise, power consumption, inearity and a well-matched input impedance (to be able to interface with the preselected filter that precedes the LNA)^21^.

Several basic structures of LNA are available in the literature and improved in several recent researches such as: Resistive terminated LNA, Inductive degenerate LNA, Resistive feedback LNA and Cascaded LNA^22–26^. The work presented in this paper is an improved architecture of the cascaded LNA.

The remainder of this paper was organized as follows. In Sect. 2, the design of the UWB cascaded LNA was presented and the theoretical study of the used technique was explained. We validated the employed technique through simulation in Sect. 3. Finally, Sect. 4 was devoted to draw some conclusions.

## THE CMOS cascaded lna design

**Figure 1:**
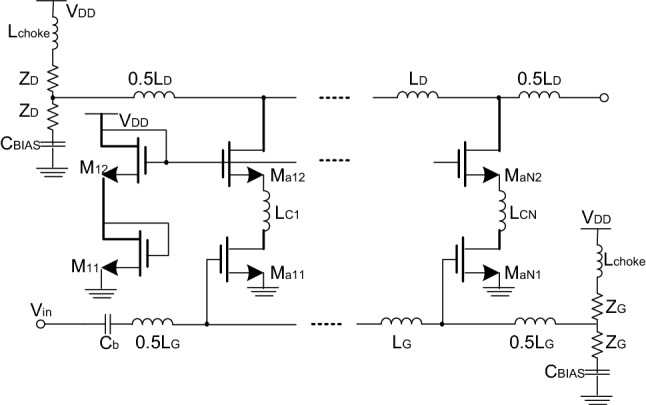
Schematic of the N-stage LNA ^27^.

The high-power consumption and large area are the two main drawbacks that have limited the cascaded amplifier application space. The resolution of these problems has become a big challenge in order to take full advantage of the intrinsic feature broadband that goes all the way down to consumed current, and the good input and output matching of the amplifier. In^27^, as shown in Fig. 1, an example of LNA is designed using several inductances, which increases the amplifier surface.

**Figure 2:**
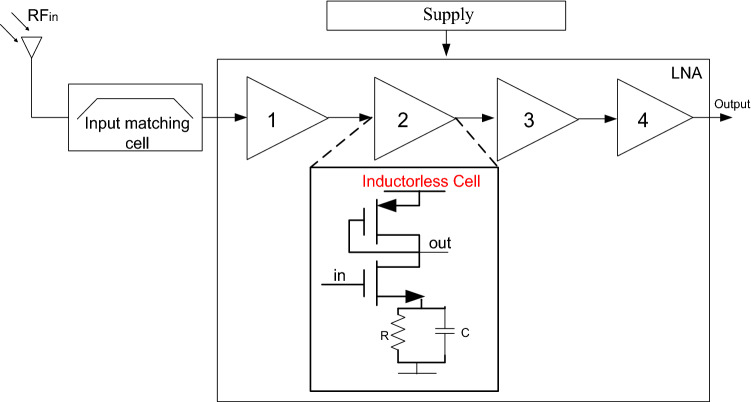
The cascaded LNA architecture.

In the proposed architecture, we have minimized the surface area of this architecture by reducing the number of inductances and involving the strategy of the cascaded stages without affecting the other performances. The proposed LNA architecture is presented in Fig. 2. It consists of matching the LNA at the input in a first step then at the output in a second step to guarantee a desired signal along the circuit and at the output.

**Figure 3:**
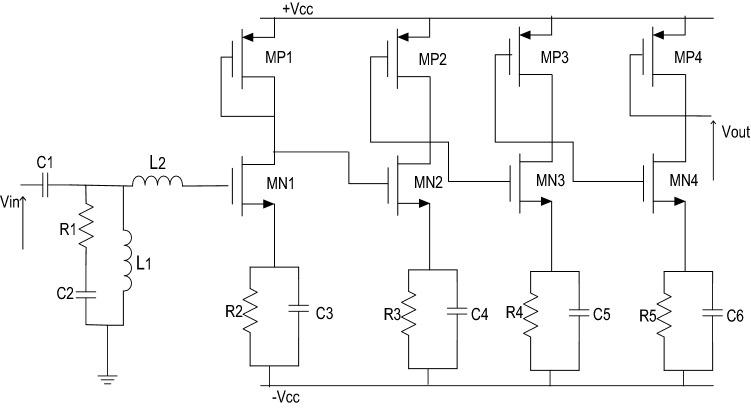
LNA transistor level.

The amplification is provided by 4 inductorless cells. The transistor level implementation of the LNA is presented by Fig. 3. It shows that the input matching circuit contains only two inductances, two capacitances and one resistance. The four amplification stages have almost the same architecture: an NMOS transistor driver with its load impedance in the form of a PMOS device. The use of both of resistors and capacitors plays a key role to get a good impedance matching and to achieve the desired bandwidth. The values of the resistors and the capacitors are respectively 0.76 kΩ and 2.2 pF. In order to further boost the performance of the amplifier, a symmetrical power supply was used. Various research studies are taking place to enable the use of symmetrical power supply in microelectronic systems^11^.

## LNA gain analysis

The LNA design requires a detailed study of its parameters^28^. The primary characteristic to be analyzed is the gain. The gain simplified equation of a one stage is given by:

1 $$G_{1s} = - gm_{n} \times R_{MP}$$

with R_MP_ is the impedance of PMOS transistor and presented by Eq. (2):

2 $$R_{MP} = \frac{1}{{\beta_{p} \left( \frac{W}{L} \right)_{p} (V_{gs} - V_{th} )_{p} }}$$

According to Eq. (1) and Eq. (2) the one stage voltage gain and the total voltage gain are given respectively by Eq. (3) and Eq. (4).

3 $$G = - \frac{{\mu_{n} }}{{\mu_{p} }} \times \frac{{\left( \frac{W}{L} \right)_{n} }}{{\left( \frac{W}{L} \right)_{p} }} \times \frac{{\left( {V_{gs} - V_{th} } \right)_{n} }}{{\left( {V_{gs} - V_{th} } \right)_{p} }}$$

4 $$G_{tot} = G \times N$$

where β is the transistor constant, N is the number of stages, µ_n_ and µ_p_ are the mobility in doped semiconductor of NMOS and PMOS transistors, respectively, (*W*/*L*)_*n*_, (*W*/*L*)_*p*_, (*V*_*gs*_ − *V*_*th*_)_*n*_ and (*V*_*gs*_ − *V*_*th*_)_*p*_ are respectively the transistors dimensions and the saturation voltages of NMOS and PMOS devices.

**Figure 4:**
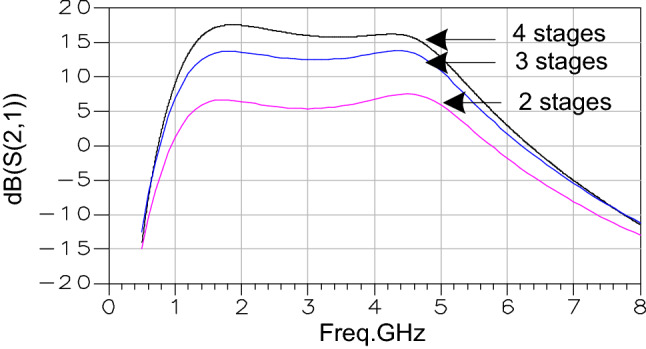
Gain comparison for different number of stages.

The Fig. 4 confirms that the gain (S(2,1)) is directly dependent on the number of stages; the more the number increases, the greater the gain will be. In addition, we note that each block offers an additional gain of 4 dB.

##  LNA noise analysis

The second characteristic is the intrinsic circuit noise. To calculate the LNA noise figure (NF), two noise types namely thermal and flicker noises are generated by MOS transistors. The noise generated by one stage is presented by Eq. (5).

5 $$NF = 4kT\left[ {\frac{2}{3}\left( {\frac{1}{{g_{mp} }} + \frac{1}{{g_{mn} }}} \right) + \frac{1}{R}} \right] + \frac{{K \times I_{d} }}{f}\left( {\frac{1}{{g_{mp}^{2} }} + \frac{1}{{g_{mn}^{2} }}} \right)$$

with k is the Boltzman constant, *T* is the temperature in kelvin, *K* is the flicker constant, *I*_*d*_ is the bias current, *f* is the bandwidth, gm is the MOS transconductance (*g*_*mp*_ for PMOS transistor and *g*_*mn*_ for NMOS transistor) and R is the resistance connected to the NMOS transistor source. We calculated the LNA total noise by relying on the Friis formula (Eq. (6)) which is used to calculate the total noise figure of the cascade stages.

6 $$NF_{tot} = NF_{1} + \frac{{NF_{2} - 1}}{{G_{1} }} + \frac{{NF_{3} - 1}}{{G_{1} G_{2} }} + \frac{{NF_{4} - 1}}{{G_{1} G_{2} G_{3} }}$$

**Figure 5:**
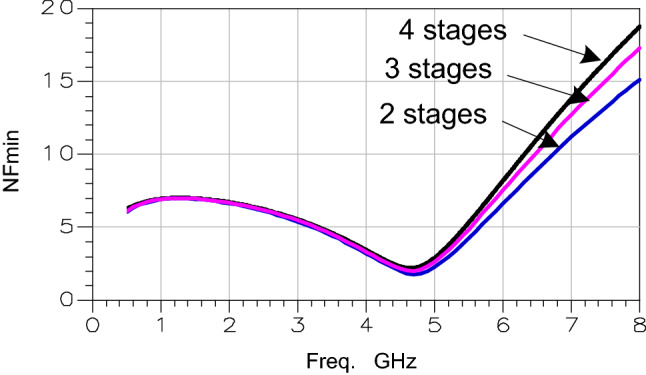
Noise comparison for different number of stages.

Since the cascaded LNA 4 stages are similar, they generate the same noise and gain. Therefore, taking Fig. 5 into consideration, the total LNA noise is primarily established by the noise figure of its first amplifying stage. The total noise figure is provided by Eq. (7).

7 $$NF_{tot} = NF + \frac{{\left( {NF - 1} \right)}}{{G^{3} }}\left( {G^{3} + G^{2} + 1} \right)$$

### Power consumption analysis

The consumed power is one of the LNA important characteristics. It should be taken into account especially for transistor sizing. The total power consumption of the cascaded LNA is equal to:

8 $$P_{dc} = N \times I \times V_{dd}$$

where I, N × I and V_dd_ are respectively the stage one current, the total current budget for the LNA and the voltage supply.

### Cascaded amplifier sizing

**Figure 6:**
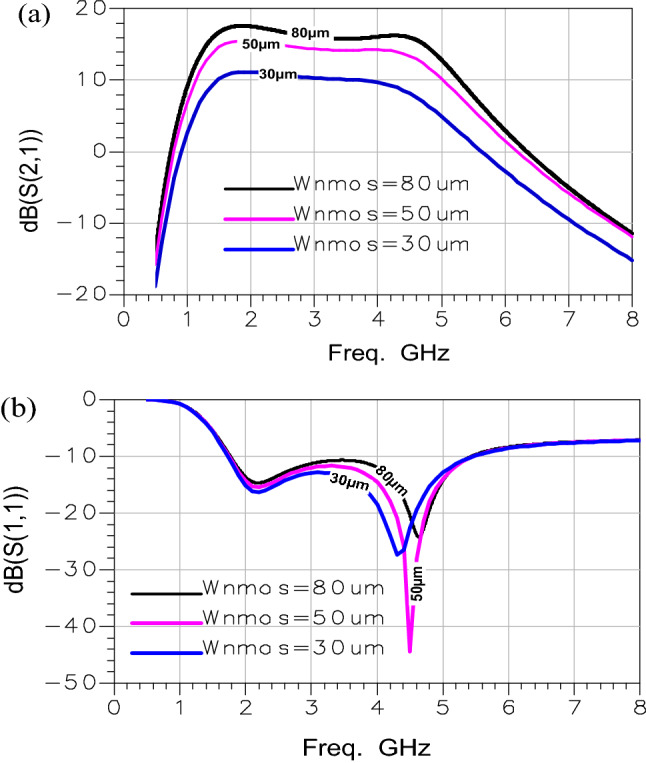
(**a**) Gain curve (S(2,1)), (**b**) Input reflection coefficient curve (S(1,1)): for different NMOS transistor width (W_NMOS_) values.

The LNA design optimization is a very important step to get a distributed amplifier with good performances. The LNA sizing including the four amplification stages is achieved as follows:

(i) First, we set the circuit specification presented by Table 1.
(ii) We established the current consumed by one stage (I) according to the above-mentioned specifications which allows calculating the PMOS transistor width. Then, we varied the NMOS transistor width for a single value of (V_gs_-V_th_)_n_ as shown in Fig. 6.
(iii) In order to satisfy the specification requirements introduced in Table 1 and obtain the optimal sizing, we spotted the second step (ii) for several values of (V_gs_-V_th_)_n_.

**Table 1:** The proposed LNA Specifications.

Parameters	Values
S(2,1) (dB)	> 15
NF(dB)	< 5
S(1,1)/S(2,2) (dB)	< -10
Pdc (mW)	< 15
IIP3 (dBm)	> 0

According to Fig. 6, we observed that the input reflection coefficient (S(1,1)) reaches its minimum value for the NMOS transistor width (W_nmos_) equal to 80 µm. Hence, if we further increase the W_nmos_ value, the S(1,1) becomes greater than -10 dB. Therefore, the W_nmos_ optimum value is 80 µm.

## Simulation results

**Figure 7:**
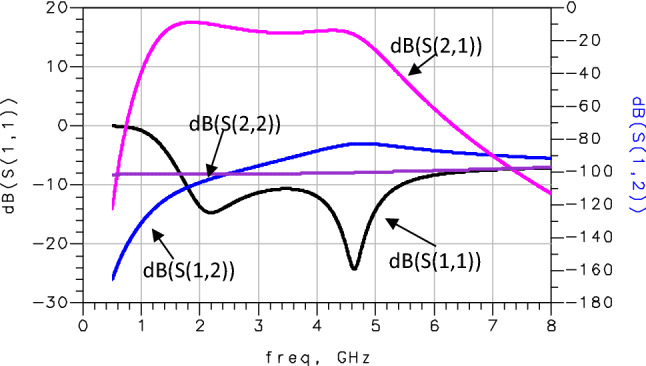
S-parameters of the proposed LNA.

The cascaded amplifier was simulated using CMOS 0.18 µm process. In this section, we validated the proposed techniques and the LNA specifications through simulation. The Fig. 7 shows the simulated LNA voltage gain (S(2,1)), the input reflection coefficient (S(1,1)), the output reflection coefficient (S(2,2)) and the reverse transmission coefficient (S(1,2)). As seen from this Figure, the LNA has a maximum gain of 17.5 dB and an S(1,2) parameter inferior to -80 dB which presents a good isolation between the input and the output of the distributed amplifier. The S(1,1) parameter is less than -10 dB and the S(2,2) parameter is lower than -8 dB. This confirms a good adaptation at the input and output of the proposed amplifier.

**Figure 8:**
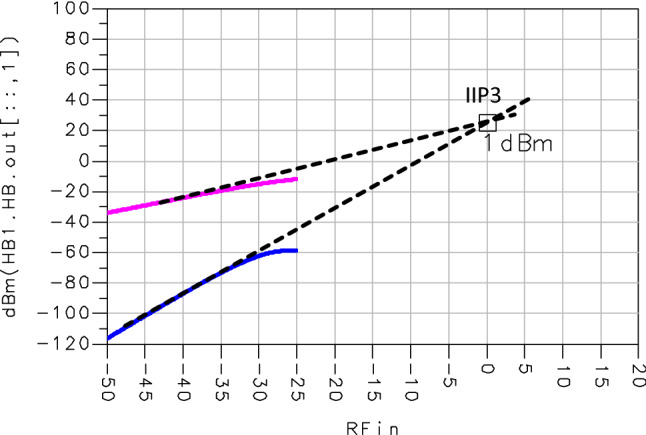
Third input intercept point (IIP3).

The LNA linearity measurement is important because it might be saturated, and this saturation leads to output power spectrum harmonics. To measure the proposed LNA linearity, we calculated the third intercept point IIP3 presented in Fig. 8 which is equal to + 1dBm. Therefore, the designed LNA provides a good linearity.

**Figure 9:**
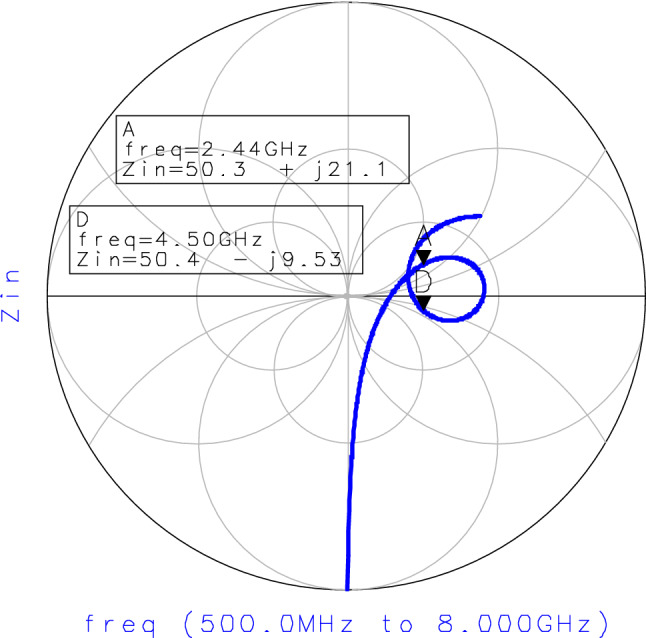
Input impedance matching curve.

The real part of the input impedance matching varies between 30Ω and 70Ω. The best adaptation (50 Ω) is performed at 2.4 GHz and 4.4 GHz frequencies as indicated in Fig. 9.

The system stability was checked by testing whether its factor K is greater than 1, and B is greater than^22–24^. These coefficients are expressed by:

9 $$K = \frac{{1 - \left| {S\left( {2,2} \right)|^{2} - } \right|S\left( {1,1} \right)|^{2} + |\Delta_{S} |^{2} }}{{2\left| {S\left( {1,2} \right)S\left( {2,1} \right)} \right|}} > 1$$

10 $${\text{B}} = 1 + \left| {{{S}}{(1,1)} } \right|^{2} - \left| {{{S}}{(2,2)} } \right|^{2} - \left| {\Delta_{{\text{s}}} } \right|^{2} >0$$

where Δ_s_ is expressed as:

11 $$\Delta_{S} = S\left( {1,1} \right)S\left( {2,2} \right) - S\left( {1,2} \right)S\left( {2,1} \right)$$

**Figure 10:**
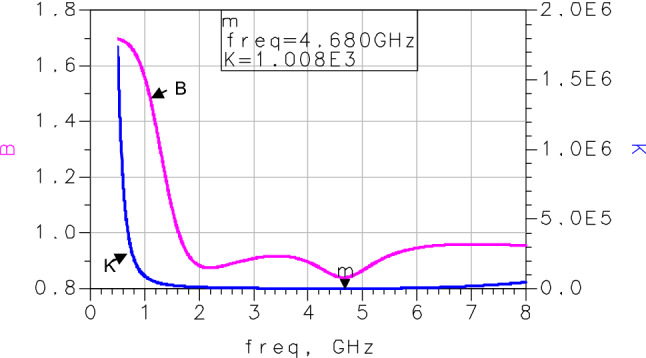
Stability coefficients K and B.

The stability coefficients (K and B) presented in Fig. 10 confirm that K is greater than 1 and B is greater than 0. Consequently, the LNA is perfectly stable.

To evaluate the performance of the designed LNA, the following Figure of Merit (*FOM*) (Eq. (12)) has been used. It combines gain (*G*), linearity (IIP3), noise figure (*NF*) and power consumption (*P*_*dc*_)^29^.

12 $$FOM = \frac{{G_{{\left( {mag} \right)}} \times IIP3_{{\left( {mW} \right)}} }}{{\left( {NF - 1} \right)_{{\left( {mag} \right)}} \times P_{{dc\left( {mW} \right)}} }}$$

The Table 2 lists the characteristics of the proposed LNA which are compared to recently published works. It is seen that the cascaded LNA has the highest FOM amongst comparable existing designs. This indicates that this circuit topology has compatibility among its features.

**Table 2:** Performance summary of UWB CMOS LNAs.

	^30^ 2018	^31^ 2019	^32^ 2016	^33^ 2009	^34^ 2006	^35^ 2013	^27^ 2010	This work
Process	45 nm CMOS	0.15 lm GaAs pHEMT/ 95 GHz	0.1 lm GaAs mHEMT/130 GHz	130 µm CMOS	130 µm CMOS	180 µm CMOS	65 µm CMOS	180 µm CMOS
Gain (dB)	14–12.8	17	22	20.47	15	8	12	17.5
BW (GHz)	24–28	17–28	18–43	0.4–10.5	DC-12	0.04–7	DC-9.5	1–5
NFmin (dB)	1.4	2.2	2.25	3.29	2.5	4.2	2.8	2
IIP3 (dBm)	4–5	N/A	N/A	-11.5	0	3	4	1
Pdc (mW)	7	30	140.4	37.8	26	9	18	13
FOM	N/A	N/A	N/A	0.02	0.28	0.34	0.61	0.65

## Conclusion

In this paper, an UWB LNA using the cascaded technique was designed. A four-stage optimized LNA was devised in the TSMC 0.18 µm CMOS process, while using only two inductances in the input matching impedance circuit. In comparison with the current works, this amplifier shows a good performances such as good gain, stability, linearity, noise and power consumption. This responds to the The trend towards miniaturization and low power consumption in the biomedical field.

## Data Availability

The datasets generated during and/or analyzed during the current study are available from the corresponding author on reasonable request.
